# Asian Immigrant Parents' Language Use and Perceptions of Parent–Child Relationship Quality

**DOI:** 10.1111/famp.70130

**Published:** 2026-02-18

**Authors:** Cindy J. Huang, Kailee Kodama Muscente, Nolan Zane, Yuuko Uchikoshi, Cindy Y. Huang

**Affiliations:** ^1^ Department of Counseling and Clinical Psychology Teachers College, Columbia University New York New York USA; ^2^ Department of Applied Psychology New York University New York New York USA; ^3^ Department of Psychology University of California Davis California USA; ^4^ School of Education University of California Davis California USA; ^5^ Department of Counseling Psychology University of Oregon Eugene Oregon USA

**Keywords:** acculturation, Asian immigrant families, language use, parent–child conflict, parent–child relationships

## Abstract

Research on Asian immigrant families has primarily focused on the influence of cultural factors, such as acculturation, on parent–child relationships, yet emerging research suggests that language use may play a critical role, especially when multiple languages (e.g., English and/or a heritage language; HL) may be used in the parent–child communication context. This exploratory study investigated the associations between parent language use and perceived parent–child relationship quality (i.e., positive relationship, parent–child conflict) among Asian immigrant parents (*N* = 90) of early adolescents ages 9–13. A MANCOVA was conducted to examine these associations, controlling for parent and child sociodemographic factors. Results indicated that parent‐reported language use was significantly associated with perceived parent–child conflict: English‐speaking parents reported higher levels of parent–child conflict (*M* = 1.65, SE = 0.29) than their HL‐speaking (*M* = 0.61, SE = 0.30) and bilingual counterparts (*M* = 0.81, SE = 0.19; *F*(2, 80) = 4.04, *p* = 0.021, partial *η* ^2^ = 0.09). When comparing the English language group against the bilingual language group, perceived parent–child conflict was statistically significant (*p = 0*.048). Parent‐reported language use was not associated with perceived positive relationships. Findings highlight language use as an important mechanism in shaping parent–child relationships in Asian immigrant families, above and beyond the effects of acculturation. Greater attention to language use may strengthen future research and family interventions aimed at improving parent–child relationships in Asian immigrant families.

## 1 Introduction

Despite extensive research dedicated to examining cultural factors in Asian immigrant families (Crane et al. 2005; Kim et al. 2009; Lee et al. 2000), language use is seldom explored as a contributing factor to immigrant family outcomes. Approximately 6.9 million Asians (i.e., East Asians, Southeast Asians, and South Asians) in the United States (U.S.) speak more than one language (Lee and Mock 2005). Many Asian immigrant families in the U.S. consist of parents who are non‐U.S.‐born (i.e., first‐generation immigrants) or parents with immigrant parentage (i.e., second‐ or later‐generation immigrants; Pew Research Center 2013). These Asian immigrant parents may choose to speak in English, a heritage language (HL; a non‐majority language spoken in or inherited from the family context; Montrul 2016; Valdés 2014), or both languages (i.e., being bilingual) with their children. Historically, bilingualism has been defined as having near‐native proficiency and equal mastery in two languages, but contemporary research has defined bilinguals as individuals who use different languages with varying levels of proficiency, in different contexts for different purposes (DeSouza et al. 2023; Wei 2000). For example, bilingual immigrant parents may use English with their children who prefer speaking in English, even if parents have limited fluency in English or typically prefer speaking only in the HL in other contexts (Wright et al. 2015).

The family context is the primary language contact setting for Asian immigrant families, with implications for parent–child relationship quality due to the cultural values and identities of the family members reflected in their language use (Álvarez‐Pérez and Harris 2022; King and Fogle 2013; Rumbaut 2008). A language contact setting (King and Fogle 2013; King et al. 2008) is defined as a setting in which two or more languages interact (De Houwer 2015). Given that English is the dominant and preferred language used in the U.S. and among youth (Cox Jr et al. 2021), immigrant parents have the unique challenge of determining which language—English, the HL, or both—to use to communicate with their children in the family context, the setting in which parents have greater influence and decision‐making power. The dynamic nature of immigrant parents' language use with their children may be due to their views of the parent–child relationship as one of the most significant socialization contexts for their children (Baquedano‐López and Mangual Figueroa 2011; Tseng and Fuligni 2000). A growing body of literature has also identified the benefits of immigrant parent bilingualism on family and child outcomes (Mueller et al. 2020; Zhang et al. 2018). Studies on Chinese immigrant mothers who are bilingual in English and the HL found that the children had fewer depressive symptoms, due to more positive parent–child communication and higher levels of youth bicultural identity formation (Liu et al. 2009; Mills 2001). This finding suggests an association between parent language use, including bilingualism, on positive parent–child relationships; however, this association still needs to be explicitly examined in the Asian immigrant family context.

Most existing studies have focused primarily on the impact of acculturation (i.e., the process of adopting the language, attitudes, and behaviors of the new host country; Zane and Mak 2003) on parent–child relationship outcomes (Bornstein and Cote 2006), rather than language use. Although language and culture are tightly intertwined, language use has frequently been investigated only in conjunction with or as a subcategory of acculturation. In the few studies that have examined Asian immigrant family language use, HL use has been found to be associated with less acculturation and more alignment with HL cultural values (Harlin and Paneque 2006), such as collectivism and the importance of communal relationships (Lee and Mock 2005; Matsumoto et al. 2008). In fact, HL use in Asian immigrant families has been shown to be associated with more family cohesion due to family members' presumptions of shared cultural values linked with HL use (Portes and Hao 2002). Alternatively, while English use may reflect higher levels of acculturation, it may also clash with Asian immigrant parents' cultural expectations of what constitutes respectful behavior and deference from their children, particularly when parents are less acculturated than their children (Portes and Hao 2002; Tseng and Fuligni 2000). These findings have been consistent across East Asian (e.g., Chinese, Korean) and Southeast Asian (e.g., Vietnamese, Hmong) families (Portes and Hao 2002; Xiong et al. 2013), indicating similar experiences of the intersection of language and cultural values between various Asian immigrant families.

However, emerging scholarship suggests that language use within immigrant families may be driving the observed effects previously ascribed to acculturation (Cox Jr et al. 2021). The phenomenon in which children of immigrants acquire English and lose HL proficiency more rapidly due to acculturation, while immigrant parents typically maintain the HL and make only modest gains in English proficiency, leads to an erosion of a shared language used between immigrant parents and children (Cox Jr et al. 2021, 2025). This shared language erosion contributes to the reduced ability to communicate effectively, which leads to increased parent–child conflict and exacerbated pre‐existing issues in the parent–child relationship due to poor communication (Cox Jr et al. 2021; Hwang 2006; Schofield et al. 2017). While acculturation may contribute to differences in language proficiency between immigrant parents and their children, language use is the primary tool for communication; the choice in language use among parents and children is not solely dictated by language proficiency (e.g., children may speak in the HL with their parents even if they are more proficient in English). The languages used between parents and children may help explain some of the inconclusive findings regarding the effects of acculturation on parent–child relationships in Asian immigrant families (Cox Jr et al. 2021; Telzer 2011). Further investigation and isolation of the effects of language use on parent–child relationships is warranted, while accounting for the impact of acculturation.

Immigrant parents' language use and perceptions of the parent–child relationship are particularly relevant to examine during the early adolescent developmental stage (roughly ages 10–13), a sensitive time period that contributes to significant relational changes (Wen et al. 2024). Early adolescents experience more unsupervised time with their peers and increased distance from their parents, which may have negative effects on the parent–child relationship (Casey et al. 2008). Parent–child conflict also tends to increase in frequency and intensity during the transition from childhood to adolescence (Barber and Olsen 2004; Laursen et al. 2017). In fact, parent–child conflict in early adolescence has been argued to be a continuation of the relationship dynamic and family linguistic context inherently stable over time, predicting the quality of the parent–child relationship into adolescence and beyond (Conger and Ge 1998; Eisenberg et al. 2008; Medvedeva 2012). Early adolescence may be a critical period to investigate and gain insight into the longstanding parent–child relationship quality from childhood into early adolescence. Exploring immigrant parents' perspectives is also critical to understanding the effects of language use on parent–child relationships during early adolescence. While both parents' and children's perspectives are important, increased understanding of parents' language use and perceptions is essential to informing parents' development in various components of parenting during this developmental stage, such as bonding, discipline, responsivity, and sensitivity to their children (Mowder 2005). Better understanding of parents' perceptions of the parent–child relationship can also improve parental engagement in child and family mental health treatment (Haine‐Schlagel and Walsh 2015).

### 1.1 The Present Study

In this exploratory study, we examined the associations between parent language use and perceived parent–child relationship quality (i.e., positive relationships, parent–child conflict) among Asian immigrant parents of early adolescents. Based on existing literature (Liu et al. 2009; Tseng and Fuligni 2000), we hypothesized that parents' HL use would be associated with more perceived positive relationships and less perceived conflict with their children, while parents' English use would be associated with less perceived positive relationships and more perceived conflict with their children. We also hypothesized that parents' bilingualism (i.e., speaking both English and the HL with their children) would be associated with more perceived positive relationships and less perceived conflict with their children.

## 2 Method

### 2.1 Participants

Participants in this study were 90 Asian immigrant parents (i.e., first‐ or later‐generation immigrant) who reported having children between the ages of 9 to 13 years old (*M*_age_ = 11.01 years). This study sample was taken from a larger study examining Asian parents' perceptions of an evidence‐based family intervention aimed at improving parent–child relationships and child behaviors. Participants were recruited with the support of a community‐based social services agency serving Asian immigrants in an urban area in northern California. Most of the participants identified as Vietnamese (40.0%), Hmong (27.8%), and ethnic Chinese born in Vietnam (i.e., Chinese‐Vietnamese; 25.6%). Participants were primarily female (74.4%) and ranged from 26 to 58 years old (*M*_age_ = 39.90 years). Most of the parents identified as first‐generation immigrants (*n* = 80), with age of immigration ranging from 1 to 48 years old (*M*_age of immigration_ = 21.83 years; SD = 12.75). Slightly over half of the participants were married (52.2%), and most of the participants (74.4%) had at least a high school diploma. Parents reported their language use with their child as English only (*n* = 16), heritage language only (*n* = 26), or a combination of the two (i.e., bilingualism; *n* = 48). Additional participant characteristics are shown in Table 1.

**TABLE 1:** Descriptive characteristics of sample (*N* = 90).

Variable	Frequency (%)
Sex (*M*_age_ = 39.90)	
Male	23 (25.6%)
Female	67 (74.4%)
Ethnicity	
Chinese‐Vietnamese	23 (25.6%)
Filipino	1 (1.1%)
Hmong	25 (27.8%)
Taiwanese	2 (2.2%)
Vietnamese	36 (40.0%)
Bi‐ethnic Chinese and Vietnamese	2 (2.2%)
Bi‐ethnic Chinese and Filipino	1 (1.1%)
Immigrant status	
U.S.‐born	10 (11.1%)
Non‐U.S.‐born (*M*_age of immigration_ = 21.83, SD = 12.75)	80 (88.9%)
Marital status	
Married	47 (52.2%)
Living together	11 (12.2%)
Separated	5 (5.6%)
Divorced	8 (8.9%)
Widowed	2 (2.2%)
Single	16 (17.8%)
Missing	1 (1.1%)
Highest level of education	
No formal education	1 (1.1%)
Elementary school (Grades 1–6)	8 (8.9%)
Junior high (Grades 7–9)	6 (6.7%)
Partial high school (Grades 10–11)	8 (8.9%)
High school diploma	24 (26.7%)
Partial college (1–3 years)	21 (23.3%)
College degree (B.A., B.S.)	16 (17.8%)
Graduate degree (M.A., M.S., Ph.D.)	6 (6.7%)
Relationship to child	
Biological mother	65 (72.2%)
Biological father	20 (22.2%)
Adoptive mother	2 (2.2%)
Adoptive father	1 (1.1%)
Grandparent	1 (1.1%)
Child grade in school (*M*_age_ = 11.01 years)	
3rd–5th grade	44 (48.9%)
6th–8th grade	44 (48.9%)
Missing	2 (2.0%)
Parent language use with child	
Heritage language	26 (28.9%)
English	16 (17.8%)
Bilingual	48 (53.3%)
Parent‐reported child language use with parent	
Heritage language	23 (25.6%)
English	33 (36.7%)
Bilingual	34 (37.8%)

### 2.2 Procedure

Participants were included in this study if they (a) self‐identified as Asian or Asian American and (b) had a child between the ages of 9–13 years old. Eligible participants were identified by the community‐based agency and consented by members of the research team. Once participants consented, they completed a survey packet in their preferred language. If participants had more than one child ages 9–13 years old, they were asked to select the oldest child in that age range and complete the survey packet based on that child. The majority of participants completed the survey packet at the community‐based agency; when the participants were unable to meet at the community‐based agency, members of the research team met with the participant at a convenient location for the participant to provide consent and complete the survey packet. This study was approved by the University of California, Davis Institutional Review Board.

### 2.3 Measures

#### 2.3.1 Demographic Variables

Participants completed a demographic questionnaire that included their age, sex, ethnicity, marital status, highest level of completed education, immigrant status, and age of immigration if applicable. Participants also answered questions regarding the child's age and grade level in school.

#### 2.3.2 Acculturation

The Vancouver Index of Acculturation (VIA; Ryder et al. 2000) is a widely used measure of acculturation to mainstream culture and enculturation to the culture of origin for Asian immigrants (Huynh et al. 2009). The VIA consisted of 20 total items, with ten questions measuring acculturation to mainstream culture (e.g., “I am comfortable interacting with typical American people”; “I believe in mainstream American values”). Items were rated on a nine‐point Likert scale ranging from 1 (*disagree*) to 9 (*agree*). A mean score for the acculturation measure was calculated, with higher scores indicating a greater endorsement of acculturation to mainstream culture. The VIA has been used with Asian adults (Hsu et al. 2012) and is significantly associated with other measures of Asian self‐identity and acculturation (Zhang and Tsai 2014). This measure demonstrated good internal reliability (*α* = 0.87).

#### 2.3.3 Perceptions of Parent–Child Relationship Quality

Perceptions of parent–child relationship quality were assessed using two measures: Positive Relationship and Parent–Child Conflict. Positive Relationship reflects positive aspects of the parent–child relationship quality, while Parent–Child Conflict reflects negative aspects of the parent–child relationship quality. The two measures have been widely used with minority families (Criss and Shaw 2005; McWhirter and McWhirter 2008; Metzler et al. 1998), including Asian families (McWhirter 2021). Mean scores for each subscale were calculated, with higher scores indicating greater endorsement of the respective parent–child relationship quality.

##### 2.3.3.1 Positive Relationship

Positive Relationship consisted of five items from the Adult‐Child Relationship Scale, which was adapted from the school‐based Student‐Teacher Relationship Scale (Pianta and Steinberg 1991). This measure assessed parents' perceptions of their relationship with their child regarding communication, level of comfort with each other, and positive feelings towards each other (e.g., “If upset, this child seeks comfort from me” or “This child is open with me about sharing feelings and telling me how things are”; Criss and Shaw 2005; Pianta and Nimentz 1991). Items were rated on a five‐point Likert scale, ranging from 1 (*definitely not*) to 5 (*definitely*). This measure demonstrated good internal reliability (*α* = 0.82).

##### 2.3.3.2 Parent–Child Conflict

Parent–Child Conflict consisted of a four‐item subscale of the Community Action for Successful Youth (CASEY; Metzler et al. 1998) instrument, developed and adapted from a National Institute on Drug Abuse‐funded intervention trial through the Oregon Research Institute (ORI) to be used in community intervention research (Biglan et al. 1994). This subscale assessed how often parents expressed anger or argued during conflict (e.g., “We got angry at each other” or “One of us got so mad, we hit the other person”). Items were rated on a seven‐point Likert scale, ranging from 0 (*never*) to 6 (*7+* *times*). This measure demonstrated good internal reliability (*α* = 0.87).

#### 2.3.4 Parent and Child Language Use

To assess parent language use with their child, participants responded to a language screening measure (Williams et al. 2019), specifically to the question “What language(s) do you use when speaking to your child?” using a five‐point Likert scale with the following items: (1) “Only English,” (2) “Only native language,” (3) “Both native language and English but more native language,” (4) “Both native language and English but more English,” (5) “Native language and English equally.” Parent language use was coded as heritage language (HL) when they selected “Only native language,” English (E) when they selected “Only English,” and bilingual (B) when they selected “Both native language and English but more native language,” “Both native language and English but more English,” or “Native language and English equally” for this item.

Participants also reported on their child's language use with them by responding to the question “What language(s) does your child use to speak with you?” using the same five‐point Likert scale. Parent‐reported child language use was coded using the same categories as above.

### 2.4 Data Analysis

Data were analyzed using SPSS version 29. Of the 100 respondents, 10 were removed due to missing sociodemographic data, which was managed using listwise deletion. Participant sex and immigrant status were dummy coded prior to analyses (participant sex: 0 = male, 1 = female; immigrant status: 0 = not born in the U.S., 1 = born in the U.S.). Both parent and child language variables were coded into three language categories: heritage language (HL) = 0, English (E) = 1, and bilingual (B) = 2. First, descriptive statistics and bivariate correlation analyses were conducted. Then, a one‐way MANCOVA was conducted to examine whether parent language use is associated with perceived parent–child relationship quality outcome variables. Preliminary analyses were conducted, including tests for MANCOVA assumptions. QQ plots and histograms were examined, and assumptions of normality were met. Heteroscedasticity was detected after fitting predictors to the residual plot, and the homogeneity of covariance matrices was evaluated using Box's test. The test was significant (*M* = 60.42, *F*(6, 22,638) = 9.64, *p* < 0.001). As preliminary tests of equality of variances are not always robust to Type I error, especially when using unequal sample sizes (Zimmerman 2004), the Wilks' lambda results were interpreted, which is more robust in unbalanced samples with heterogeneous variance (Ateş et al. 2019). Demographic control variables for parent (age, sex, highest level of completed education, immigrant status, acculturation) and child (age and parent‐reported child language use with parent) were entered as covariates. Positive relationship or parent–child conflict were entered as outcome variables. Post hoc pairwise comparisons using Bonferroni correction were conducted to examine differences between parent language groups on parent–child relationship quality outcomes.

## 3 Results

### 3.1 Descriptive Statistics

Table 2 reports the descriptive statistics and bivariate correlations among the primary variables of interest. Means demonstrated that the English language use comparison group reported less perceived positive relationships than the other language use groups (English: *M* = 2.84; HL: *M* = 3.29; bilingual: *M* = 3.36). Means also demonstrated that the English language use comparison group reported higher rates of perceived parent–child conflict (English: *M* = 1.70; HL: *M* = 0.36; bilingual: *M* = 0.93).

**TABLE 2:** Bivariate correlations among continuous variables of interest in overall sample (*N* = 90).

Variable	*M*	SD	1	2	3	4	5	6	7
1. Age	39.90	6.78	—						
2. Sex^a^	0.74	0.44	−0.13	—					
3. Immigrant status^b^	0.19	0.39	−0.36***	0.09	—				
4. Education	5.26	1.69	−0.30**	−0.05	0.35***	—			
5. Child's age	11.01	1.54	0.32**	0.04	−0.28**	−0.17	—		
6. Acculturation	5.81	1.94	−0.28**	−0.06	0.41***	0.48***	−0.11	—	
7. Positive relationship	3.25	0.72	−0.17	0.28**	−0.02	−0.09	−0.07	−0.09	—
8. Parent–child conflict	0.90	1.20	−0.30**	−0.03	0.21*	0.21*	−0.20	0.12	−0.27**

^a^ Sex: 0 = male, 1 = female.

^b^ Immigrant status: 0 = not born in the U.S., 1 = born in the U.S.

* *p* < 0.05.

** *p* < 0.01.

*** *p* < 0.001.

### 3.2 Parent Language Use on Perceived Parent–Child Relationship Quality

**FIGURE 1:**
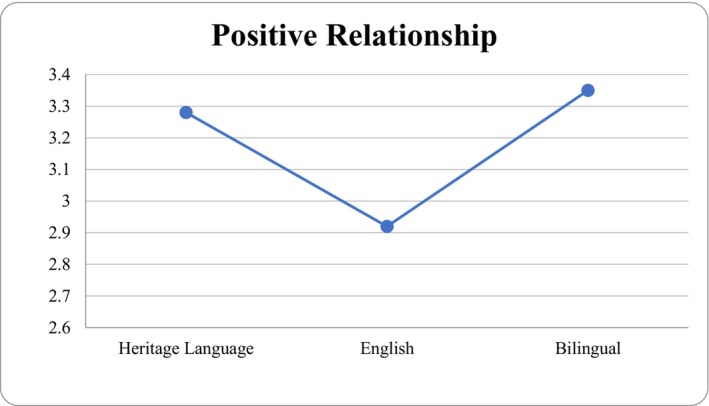
Estimated marginal means for parent language use by positive relationship.

**FIGURE 2:**
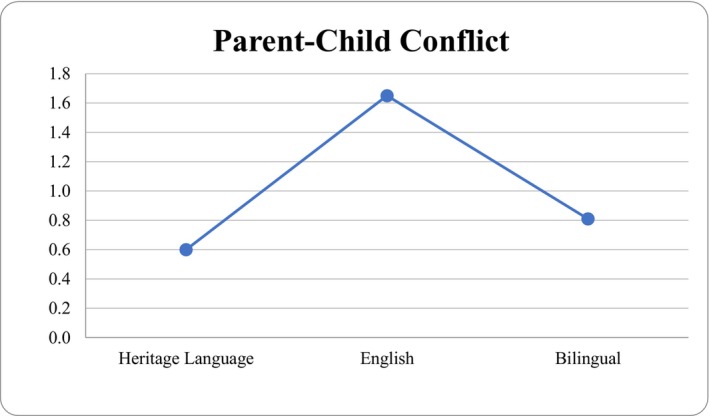
Estimated marginal means for parent language use by parent–child conflict.

Significant findings emerged for the one‐way MANCOVA assessing the effects of parent language use on perceived parent–child relationship quality (*F*(2, 79) = 2.66, *p* = 0.035, Wilks' *Λ* = 0.878, partial *η* ^2^ = 0.06). Estimated marginal means indicated that English‐speaking parents reported higher levels of perceived parent–child conflict (*M* = 1.65; SE = 0.29) than their HL‐speaking (*M* = 0.61; SE = 0.30) and bilingual counterparts (*M* = 0.81; SE = 0.19; *F*(2, 80) = 4.04, *p* = 0.021, partial *η* ^2^ = 0.09; see Table 3). Pairwise comparisons indicated that parent perceived parent–child conflict was statistically significant when comparing the English language use group against the bilingual language group (*p* = 0.048). There were no statistically significant differences in perceived parent–child conflict between the bilingual and HL groups. Parent language use was not significantly associated with perceived positive relationships. See Figures 1 and 2 for the estimated marginal means of parent language use by perceived positive relationship and parent–child conflict.

**TABLE 3:** Estimated marginal means, standard errors, and univariate results for parent language use on positive relationship and parent–child conflict.

	Heritage Language		English		Bilingual		*F*(2, 79)	*η* ^2^
	*M*	SE	*M*	SE	*M*	SE		
Positive relationship	3.19	0.19	2.91	0.18	3.39	0.12	2.62	0.061
Parent–child conflict	0.61	0.30	1.65	0.29	0.81	0.19	4.04*	0.092

*Note:* Covariates for the parent (age, sex, immigrant status, education, and acculturation) and the child (age and parent‐reported child language use) were included.

* *p* < 0.05.

## 4 Discussion

This exploratory study examined the associations between Asian immigrant parents' language use and perceptions of their parent–child relationship quality with their early adolescent children. Findings indicated that parent language use was associated with perceived parent–child conflict—specifically, English‐speaking parents reported more perceived parent–child conflict with their children than their HL‐speaking or bilingual counterparts. This finding partially supports our hypotheses and is consistent with existing research on the association between English use and increased parent–child conflict in Asian immigrant families (Portes and Hao 2002; Tseng and Fuligni 2000). Given that the average age of immigration for immigrant parents in this sample was about 22 years, with most parents reporting having at least a high school diploma, some of these parents may have had very different educational experiences from their children in the U.S. (Fuligni and Fuligni 2007). These linguistic and cultural differences could increase parent–child conflict when parents are communicating in English. Due to differing cultural and linguistic norms, immigrant parents who report speaking English with their children may perceive more misunderstandings and miscommunication, leading to more perceived parent–child conflict (Cox Jr et al. 2021). On the other hand, HL‐speaking parents may perceive more deference from their children and more harmonious relationships when using the HL with their children, which may lead to less perceived parent–child conflict compared to English‐speaking parents (Harlin and Paneque 2006). Furthermore, bilingual parents may be able to communicate more effectively with their children by switching between English and the HL with more cognitive flexibility (Marzecová et al. 2013; Mouw and Xie 1999), thus contributing to less perceived parent–child conflict.

Findings also indicated that parent language use was not associated with perceived positive relationships. While this result may be due to limited power and uneven groups, perceived parent–child conflict may be more salient during early adolescence, compared to perceptions of a positive parent–child relationship. During this developmental stage, parents and adolescents are renegotiating their relationship while navigating the adolescents' desire for more autonomy and independence (Barber and Olsen 2004; Tseng and Fuligni 2000). Parents' displays of warmth may decline temporarily during this time as parent–child conflict becomes more intense (Silva et al. 2020). Furthermore, early adolescents are likely seeking more social support from their friends and peers during this developmental stage (Yeh et al. 2008), which may make it difficult for parents to recall or report on their adolescents' behaviors that would indicate a positive relationship (e.g., seeking parental comfort, sharing feelings). Alternatively, another approach to interpreting the non‐significant finding may be that family conflict could only arise within the context of close, secure relationships that encourage open conversation and communication, whereas a lack of conflict could indicate apathy, indifference, or more distant relationships (Cummings and Schatz 2012). Future research could instead explore how relational security and specific communication practices are associated with parent–child relationships in Asian immigrant families, rather than conceptualizing parent–child conflict and positive relationships as inverts or opposite ends of a dichotomy.

Moreover, the items measuring positive relationships in this study may lack the cultural nuances necessary to accurately identify the closeness and warmth as perceived by Asian parents. The positive relationship measure focused on children seeking comfort from their parents or verbally sharing positive feelings with one another (e.g., “If upset, this child seeks comfort from me” or “This child is open with me about sharing feelings and telling me how things are”). These items may not fully capture the closeness and warmth that some Asian immigrant parents convey through instrumental support and actions rather than words (e.g., providing food or monetary support, demonstrating parental sacrifice, sharing strong parental desires for children to succeed; Russell et al. 2010; Wang et al. 2021), while balancing promoting their early adolescents' developing autonomy and individuality (Cheah et al. 2013). The non‐significant finding warrants further exploration in future research to critically examine whether contemporary measures of positive parent–child relationships are reflective of the expressions of love and affection within Asian families, particularly when parents invoke bicultural expressions of love in the parent–child relationship context (Williams et al. 2024).

These findings highlight the importance of exploring language use in the Asian family context, given the lack of significant associations between acculturation and parent–child relationship quality. In line with Cox Jr et al. (2021), recent research has shown that difficulties in effective communication, such as through language use, may contribute to poorer parent–child relationships, rather than the well‐documented effects of acculturation discrepancies or cultural values. However, findings from this study may require more research to substantiate these associations, due to the dynamic nature and bidirectional influence of parent–child language use and relationships. Future research would benefit from exploring the dyadic and reciprocal effects of language and parent–child relationship quality, while controlling for acculturation when relevant.

## 5 Limitations, Implications, and Conclusions

Due to the exploratory nature of this study, there are limitations that warrant attention. First, the data for this study was cross‐sectional and limited to parent self‐report. Future studies could investigate the impact of parent language use on perceptions of parent–child relationship quality over time and across children's developmental stages, while also incorporating child‐report data, behavioral data, and qualitative or observational data of parent–child interactions to complement parent self‐report data. This study was also limited to collapsing various Asian ethnic groups into one racial group. Future studies could investigate intergroup differences among specific Asian subgroups (e.g., between Vietnamese and Hmong families, or between East Asian and Southeast Asian families) for the variables of interest. This study did not recruit equal‐sized sub‐samples of participants based on language use, and there may be a systematic reason for the missing covariate values for the ten respondents removed due to missing data. Finally, language proficiency was not measured; given that about a quarter of our sample (26%) immigrated before the age of 12, some parents may have experienced heritage language attrition or language loss into adulthood. Future studies would benefit from utilizing standardized measures of language proficiency in analyses. This study also collapsed multiple groups into the “bilingual” group to match our definition of bilingualism, and this group may include individuals who have only adopted idiosyncratic expressions from one language to another, thus reducing important variation. These limitations may preclude generalizability among larger Asian immigrant populations.

Despite these limitations, this study has multiple noteworthy strengths. First, this study recruited an understudied, community sample of Asian ethnic populations, such as Vietnamese, Hmong, and ethnically Chinese from Vietnam (termed “Chinese‐Vietnamese” in our study). This study also focuses on a unique immigrant family communication context through language use. This focus on language use is a significant contribution to the investigation of family dynamics through parent–child communication, which has been argued in recent research to be more proximal to parent–child relationship quality than parent–child acculturation differences (Cox Jr et al. 2021).

The findings from this exploratory study demonstrate promise in understanding associations between language use and parent–child relationship quality among Asian immigrant parents and their children, a largely understudied area of research. To build upon this study, future studies can examine both parent and child language proficiency and language use to determine whether these factors impact the dyadic parent–child relationship quality. Future studies could also investigate how the relationship dynamic may drive parent–child language choices, given existing evidence demonstrating how parent–child emotions drive language use and choice (Williams et al. 2020). This could warrant more parent–child dyadic research examining language proficiency, use, and choice, as well as how parents and children switch languages in their interactions, otherwise termed codeswitching (Williams et al. 2019). Future studies can also examine the discrepancy between parent–child perceptions of the parent–child relationship quality as a result of either reciprocal or nonreciprocal language use.

This study also has noteworthy implications for clinical interventions to better understand, engage, and serve Asian immigrant families. Clinicians working with Asian immigrant families can assess parents' language use and their perceived parent–child relationship quality with their children as part of the intake assessment. Research has shown that learning about immigrant parents' perspectives can greatly increase their children's access to mental health treatment (Wang et al. 2019). For example, during treatment planning, engaging Asian immigrant parents in improving their communication and parent–child relationship quality with their early adolescents as a treatment goal may serve as a motivating factor for increasing both parents' and adolescents' engagement in family therapy (Haine‐Schlagel and Walsh 2015; Wang et al. 2021). Treatment providers could also demonstrate more awareness of linguistic barriers or communication differences to better engage Asian immigrant parents in treatment, such as using interpreter services as needed (Brassart et al. 2017). This study builds upon existing research regarding the need to better understand how Asian immigrant parents' language use contributes to their perceptions of parent–child relationship quality. Increased understanding of the associations between language use and parent–child relationships, as well as an awareness of the impact of cultural factors such as acculturation, can help improve family relationships and overall well‐being in Asian immigrant families.

## Funding

This study was supported by the UC Davis Clinical and Translational Science Pilot Program through grant #PSYRF63, awarded to Cindy Y. Huang and Nolan Zane. The pilot program for this study was funded by the National Center for Advancing Translational Sciences, National Institutes of Health, through grant number UL1 TR000002.

## Conflicts of Interest

The authors declare no conflicts of interest.

## Data Availability

The data that support the findings of this study are available from the corresponding author upon reasonable request.
